# Metagenomic evidence clarifies the texture-dependent cascading effects of organic degradation on soil hypoxia and N_2_O emission

**DOI:** 10.3389/fmicb.2025.1670657

**Published:** 2025-09-22

**Authors:** Jialin Hu, K. Taylor Cyle, Wenqiao Yuan, Wei Shi

**Affiliations:** ^1^Department of Crop and Soil Sciences, North Carolina State University, Raleigh, NC, United States; ^2^Department of Soil and Crop Sciences, Texas A&M University, College Station, TX, United States; ^3^Department of Biological and Agricultural Engineering, North Carolina State University, Raleigh, NC, United States

**Keywords:** microbiome, soil pore size distribution, anaerobiosis, soil texture, GHG emissions

## Abstract

**Introduction:**

Soil pore-scale aeration is a crucial yet often overlooked factor influencing the effectiveness of nitrous oxide (N_2_O) emission mitigation strategies. Our previous work revealed a hundred-fold variation in N_2_O emissions among soils under apparently aerobic conditions and texture-dependent mitigation effects of biochar–manure co-compost (BM) compared to manure compost (M).

**Methods:**

We analyzed soils of three textures—clay loam (CL), silt loam (SL), and sand (SA)—amended with BM or M. Metagenomic sequencing was used to profile microbial community composition and functional genes, with a focus on aeration-sensitive taxa and pathways.

**Results:**

We demonstrate that these changes of N_2_O emissions are aligned with variations in aeration-sensitive microbes and genes. SA, with the highest N_2_O emissions, was most abundant in obligate and facultative anaerobes and denitrification-related genes, while CL, with the lowest emissions, had more genes related to fermentation and dissimilatory nitrate reduction. Compared to M, BM in CL favored genes for microbial processes requiring a more reducing environment, likely because biochar-induced finer pores, exacerbating oxygen diffusion limitations. This severe oxygen restriction in CL after BM addition was substantiated by greater reductions in CO_2_ efflux and C-cycling genes than in the other soils.

**Discussion:**

Our findings suggest that hypoxic pore abundance and the severity of pore anaerobiosis imparted by degradation of organic amendments varied with soil texture and are the overriding factors of soil greenhouse gas (GHG) emissions. Metagenomic traits provide a sensitive tool for detecting pore-scale environmental shifts, improving our mechanistic understanding of soil-dependent GHG emissions following organic amendments.

## 1 Introduction

It is a daunting challenge to mitigate soil N_2_O emission despite a considerable understanding of responsible microbes, processes and conditions (Bakken et al., 2012; Barnard et al., 2005; Butterbach-Bahl et al., 2013; Zhu et al., 2013). The emission of N_2_O has shown a continued rise in the past four decades (1980–2020), primarily through agricultural practices such as nitrogen fertilization and waste recycling (Cui et al., 2024; Tian et al., 2023). While agricultural management has been reformed, emphasizing not only productivity but also environmental sustainability, the N_2_O emission from the arable land remain unabated, suggesting overlooked ecological factors limit the translation of mechanistic understanding into effective mitigation strategies.

One such factor is soil pore-scale aeration, which directly governs oxygen availability. Pore size and network control both the movement of gases within the soil matrix and across the soil-atmosphere interface (Bahlmann et al., 2020). Pore architecture may dominate over other soil properties (e.g., soil moisture) to influence N_2_O emissions (Kim et al., 2022; Pulido-Moncada et al., 2024). Often, high microporosity can create oxygen shortage through severe tortuosity of gas diffusion by capillary-filled water and therefore favor denitrification and N_2_O production (Groffman and Tiedje, 1989; Zaman et al., 2012), while high macroporosity can also lead to drastic emissions of N_2_O under certain circumstances (Kim et al., 2022; Kravchenko and Guber, 2017). When macropores are enriched with organic substances (e.g., root exudates or soil amendments), known as “hot moments,” the initially rapid decomposition will deplete oxygen, making pores oxygen-limited and conducive niches for N_2_O production. Such a cascading impact underscores the potential risk of unintended N_2_O emissions from management practices designed to enhance soil carbon sequestration, including organic amendments (Chen et al., 2013).

Soil pore heterogeneity also shapes microbial diversity, composition, and interactions (Carson et al., 2010; Hassink et al., 1993; Li et al., 2024a; Wolf et al., 2013; Xia et al., 2023; Xia et al., 2022). Distinct microbial groups may preferentially inhabit pores of different sizes; for example, alpha- and beta-Proteobacteria and Bacteroidetes are likely more abundant in large pores, whereas Actinobacteria and Chloroflexi may be favored in relatively smaller pores (Hemkemeyer et al., 2018; Seaton et al., 2020; Xia et al., 2020). The underlying drivers are implicitly attributed to variations of resource allocation (e.g., carbon and nutrients) and environment (e.g., pH, water, and oxygen) at the pore scale. While soil pore heterogeneity can be gauged or predicted by, for example, water retention curve and X-ray computed tomography (Helliwell et al., 2013; Jiang et al., 2025; Lawrence, 1977; Pires et al., 2020), pore-scale variations in resource availability and environmental conditions cannot be easily quantified, leaving uncertainty about how these microenvironments translate into whole soil N_2_O fluxes. Since microbes reside in pores and are within the immediate range of pore-scale environmental influences, their community traits could serve as reliable indicators of pore hypoxic status, nutrient availability, and associated denitrification potential (Højberg et al., 1994; Jin and Sengupta, 2024; Khalil et al., 2004).

To address this gap, we used soils of different textures to create systematic differences in pore architecture and aeration, as fine-textured soils generally have more numerous but smaller pores compared to coarse-textured soils. Compost inputs were applied to induce oxygen consumption through microbial respiration (Kravchenko and Guber, 2017; Krull et al., 2001; Negassa et al., 2015; Robertson and Paul, 2000). Chicken manure was selected in this study as the compost substrate because it is one of the most abundant livestock wastes in the United States and is rich in nitrogen that contributes substantially to N_2_O emissions. Biochar was added as a co-composting agent due to its high porosity and capacity to modify pore size distribution, aeration, and nutrient retention, making it a promising strategy for mitigating greenhouse gas emissions (Mujtaba et al., 2021; Umair Hassan et al., 2024).

Although soil texture and amendment properties such as nutrient content and porosity can influence microbial community composition, our study focuses on how microbial traits can serve as proxies for pore-scale oxygen availability, which cannot be directly measured. Our previous work demonstrated that biochar-manure co-compost mitigated N_2_O emissions compared to manure compost alone, but its effectiveness hinged on soil texture, with the strongest effects on CO_2_ in fine-textured clay loam and on N_2_O in coarse-textured sand (Hu et al., 2024; Yuan et al., 2017). Building on these findings, we hypothesize that soil texture-specific pore size distribution regulates pore-scale oxygen availability, which in turn drives distinct microbial community structures and functional gene profiles associated with organic carbon decomposition and nitrogen transformations, ultimately leading to texture-dependent N_2_O emissions. To test this hypothesis, we conducted metagenomic analysis of soils treated with manure compost (M) and biochar-manure co-compost (BM) across different textured soils. Specifically, our objectives were to (1) characterize microbial community composition and functional gene profiles associated with carbon decomposition and nitrogen transformations under different treatments and soil textures; (2) use these microbial traits as proxies to infer pore-scale oxygen availability; and (3) evaluate whether such microbial indicators can explain texture-dependent differences in N_2_O emissions.

## 2 Materials and methods

### 2.1 Microcosm experiment and greenhouse gas efflux

A 107-day laboratory microcosm experiment was conducted to assess the effectiveness of soil microbiome as an indicator to reflect soil pore-scale aeration status. Soils of three texture classes (clay loam, silt loam, and sand) were selected to provide contrasting pore size distributions that influence gas diffusion and aeration. Surface soils (0–10 cm) were collected in October 2021 from forested areas at two research stations in North Carolina, USA. Clay loam (Georgeville clay loam, Fine, kaolinitic, thermic Typic Kanhapludults; 32% sand, 41% silt, 28% clay) and silt loam (Herndon silty loam, Fine, kaolinitic, thermic Typic Kanhapludults; 29% sand, 58% silt, 14% clay) were sampled from Breeze Farm (36°09′42″N, 79°06′29″W) in the Piedmont region. Sandy soil (Candor sand, Sandy, kaolinitic, thermic Grossarenic Kandiudults; 89% sand, 8% silt, 3% clay) was collected from the Sandhills Research Station in Jackson Springs (35°10′59″N, 79°40′39″W). After collection, soils were sieved (<2 mm) to remove debris and stored at 4 °C until use in microcosm experiments.

Two organic amendments that differed not only in biodegradability but also in pore size distribution were used, including chicken manure compost (M) and biochar-chicken manure co-compost (BM, 20% of biochar generated at high temperature). Briefly, the biochar was produced from pine woodchips (Newton County, NC, USA) using top-lit updraft gasification at an airflow of 20 L min^−1^ and a peak temperature of 840.5 °C. Chicken manure compost was prepared from manure and bedding materials collected from the Department of Poultry Science, NC State University and stored in sealed containers at 4 °C until the initiation of the composting process. Biochar–manure co-compost (BM) was generated by adding 20% biochar (v/v) to chicken litter. All composts were produced from ~18 kg of raw material in ~140 L FCMP outdoor IM4000 dual-chamber tumbling composters (Ontario, Canada), which are equipped with aeration holes and deep fins to facilitate aerobic composting. The composting materials were turned two to three times daily to maintain an aerated and fluffed state. The turning accelerated the composting process, as the composting materials could reheat themselves. The temperature of the composting materials was monitored daily and exhibited a 2-day mesophilic, 1–2 week thermophilic (50–60 °C), and the rest mesophilic fluctuations throughout the 4-week composting process.

The M- and BM-amended soils (~20 g dry weight equivalent) were packed into 135 mL glass jars for the incubation experiment. In detail, each soil was amended with 5% (w/w) M or BM and packed to 1.10 g cm^−3^ (CL and SL) and 1.45 g cm^−3^ (SA) to create six treatments (2 organic amendments × 3 soils), each with three replicates. The 5% application rate was selected as a low but effective level within the range (1–10% or higher) commonly reported in compost and biochar incubation studies (Frimpong et al., 2021; Masmoudi et al., 2018; Yuan et al., 2017), ensuring detectable effects on microbial activity and greenhouse gas fluxes while remaining representative of practical soil amendment levels.

After adjusting soil water content to 60% water-filled pore space, microcosms were incubated at 25 °C for 3.5 months with periodic water addition to replenish evaporative losses and maintain soil moisture over the incubation. Since the volume of water added was minimal, soil porosity was not substantially altered. Water was always added immediately after gas sampling and/or jar aeration to prevent short-term disturbance of gas flux measurements. The incubation was terminated after 3.5 months because cumulative CO_2_ emissions had reached a plateau, indicating that microbial respiration had slowed markedly and that the added organic substrates were largely decomposed. Soil effluxes of CO_2_ and N_2_O were measured at day 1, 2, 3, 4, 5, 6, 7, 9, 11, 14, 17, 21, 25, 28, 35, 42, 64, 71, 78, 81, 85, 92, 99, and 107. The 18 jars (i.e., 6 treatments × 3 replicates) used for the collection of soil samples after 107 days of incubation were used to repeatedly measure CO_2_ and N_2_O fluxes over the incubation period. The jars were aerated by letting their lids open for 30 min after gas sampling on the measurement days. During the sampling interval days, the jars were aerated for 30 min daily during the initial three weeks of incubation and were aerated every two to three days during the fourth to ninth weeks of incubation. From the tenth week to the end of incubation, aeration was only implemented after gas sampling. Gas concentrations were quantified using a gas chromatography system with μECD detector (Agilent Technologies, PA, USA) for N_2_O and LI-870 CO_2_/H_2_O Analyzer (LI-COR, Lincoln, NE, USA) for CO_2_. A summary of greenhouse gas emissions on days 4, 21, and 107 across six treatments is shown in Supplementary Figure S1. The N_2_O and CO_2_ flux was calculated using the following equation:


$$F=\frac{(C_{\text{sample}}-C_{\mathrm{air}})\times P\times V\times M}{R\times T\times t\times m\times 1000}$$

Where *F* is the flux of N_2_O or CO_2_ (mg N kg^−1^soil h^−1^); *C_sample_* and *C_air_* are the gas concentrations in the jar headspace and ambient air (ppb), respectively; *P* is the air pressure in the jar, assumed to be 1 atm (101.325 Kpa); *V* is the total volume of the jar headspace plus the free pore volume (cm^3^); *M* is the molar mass of the gas (g mol^−1^); *R* is the ideal gas constant (8.31432 J mol^−1^ K^−^1); *T* is the jar air temperature (298 K); *t* is the measurement time (h); and *m* is the dry weight of soil (g) (Hu et al., 2024).

For shotgun metagenomic sequencing, soil samples were collected on days 4, 21, and 107 days of incubation, with 18 incubated jars (representing six treatments with three replicates) being taken from a total of 54 incubated jars (i.e., 6 treatments × 3 replicates × 3 sampling times) at each sampling time. The three sampling times represent rapid degradation of organic material, peak of cumulative CO_2_ or N_2_O emissions, and late-stage stabilization, respectively.

### 2.2 DNA extraction, shotgun metagenomic sequencing, and bioinformatics

Metagenomic DNA was extracted from ~ 0.25 g soil-compost mixture using DNeasy PowerSoil Kit (Qiagen, Germany) according to the manufacturer’s protocol. After quantification using NanoDrop One spectrophotometry (NanoDrop Technologies, Wilmington, DE), DNA was stored at −20 °C before library preparation. Due to financial constraints, replicates were composited, resulting in a total of 18 samples (3 soils × 2 composts × 3 sampling times). Libraries were prepared and shallow-depth sequenced (~ 40 million paired-end reads) on Illumina NovaSeq platform (150 bp × 2) by the Genome Sciences Laboratory, NCSU.

On average, 83.2 million raw reads per sample were obtained (range: 59.5–148.4 million reads) (Supplementary Table S1). Variations among samples were moderate, with ~ 32% of the coefficient of variation. After quality assessment with FastQC (Andrews, 2010), reads were trimmed by Trimmomatic (Bolger et al., 2014) to remove adapters and to filter out low-quality reads with “N” base and short reads < 100 bp. Approximately 99% of raw reads passed quality control and were ~ 141 bp in length. These reads were then subjected to the taxonomy analysis using KrakeN_2_ (Wood et al., 2019) and the functional gene examination using two pipelines: (1) blast of short reads against reference databases and (2) gene prediction and annotation following short reads assembly.

For the BLAST pipeline, the local BLASTx (Altschul et al., 1990) was used to align the trimmed clean reads of each sample against functional gene databases of nitrification and denitrification: ammonia-oxidizing archaea (AOA) *amoA*, ammonia-oxidizing bacteria (AOB) *amoA*, *nirK*, and *nosZ* with an E-value < 1 × 10^−5^. The relative abundance of functional genes was normalized as reads per kb per genome equivalent (RPKG), which was calculated as RPKG = (reads mapped to gene)/(gene length in kb)/(number of genome equivalents). The number of genome equivalents was estimated by MicrobeCensus (Nayfach and Pollard, 2015).

For the assembly and gene annotation pipeline, *de novo* assembly via succinct de Brujin graph approach was performed using the MEGAHIT assembler (Li et al., 2015). Then, the contigs (≥ 1,000 bp) were analyzed for open reading frames (ORFs) prediction using MetaGeneMark (Zhu et al., 2010). Non-redundant gene catalog (i.e., unigenes) was constructed with predicted ORFs using CD-HIT (Li and Godzik, 2006) at 95% identity and 90% coverage. All high-quality reads were aligned against the unigenes via Bowtie2 (Langmead and Salzberg, 2012) and Samtools (Danecek et al., 2021) to obtain the gene abundance in each sample. The relative abundance of each unigene in a sample was calculated as read counts per kilobase million (CPM), by the equation: $a_{i}=\frac{\frac{x_{i}}{L_{i}}}{\sum_{j}\frac{x_{j}}{L_{j}}}$ (Le Chatelier et al., 2013), where $a_{i}$ is CPM of gene *i*; $\;x_{i}$ is the read counts of gene *i*; $L_{i}$ is the length of gene *i*; and the denominator is the sum of all mapped reads (genes) normalized to respective gene lengths.

The annotation of unigenes was conducted by both KEGG GhostKOALA (Kanehisa et al., 2016) and eggNOG (Huerta-Cepas et al., 2019) to reveal gene orthology and ontology information, respectively. The raw sequencing data can be accessed from NCBI Sequence Read Archive (SRA) Database, BioProject No. PRJNA1165301.

### 2.3 Quantitative PCR for N-cycling genes regulating N_2_O efflux

The samples were also quantified for the abundance of nitrification and denitrification functional genes, including AOA *amoA*, AOB *amoA*, *nirK*, *nirS*, and *nosZ*. Given the prevalence of *nirK*-type denitrifiers over *nirS*-type denitrifiers in our samples and the constraints associated with conventional primer pairs (such as bias, coverage, and specificity) used for amplifying prokaryotic *nirK* genes, we employed novel primers to target various prokaryotic *nirK* clades. This approach was undertaken to provide a more comprehensive understanding of which specific clade(s) of *nirK* were predominantly involved in nitrite reduction under soil treatments.

The qPCR was performed on a CFX96 Optical Real-Time Detection System (Bio-Rad, Laboratories Inc., Hercules, CA, USA). The 20-μl qPCR reaction mixture contained 10 μL Maxima SYBR Green qPCR Master Mix (2X) (Thermo Scientific, USA), 1 μL PCR forward and reverse primer (both 10 μM), 2.5 μL DNA template, and 5.5 μL nuclease-free water. Primer sets and reaction parameters are listed in Supporting Information (Supplementary Table S2).

### 2.4 Statistical analysis

Two-way ANOVA was applied to test the effects of soil texture, compost type, and their interaction on the relative abundance of functional genes related to oxygen response, C degradation, N cycling, and antioxidant resistance. Log-transformation was conducted for non-normally distributed data. The impacts of soil texture and compost type on soil microbial communities were tested by permutational multivariate analysis of variance (PERMANOVA) based on Bray-Curtis distance matrix and visualized by non-metric multidimensional scaling (NMDS) conducted in R with packages vegan (v 2.5–7), phyloseq (v 1.38.0), and ggplot2 (v 3.3.5). DESeq analysis with DESeq2 (v 1.34.0) was also conducted to evaluate differences in the relative abundance of KEGG genes between M and BM (i.e., |log_2_-fold change| > 1). Correlation analysis was performed by Spearman rank correlation coefficient on SPSS as this nonparametric method is robust to non-normal data distributions and more appropriate than Pearson’s correlation for ecological and metagenomic datasets. Statistically significant difference level at *p* < 0.05 was used in this study unless otherwise noted.

## 3 Results

### 3.1 Microbial diversity and anaerobic microbial taxa in response to soil texture and amendment

On average, ~25% of high-quality reads were classified into microbial superkingdoms, with 99% of classified reads belonging to bacteria and 1% to fungi, archaea, and viruses (Supplementary Table S3). Shannon diversity index was significantly lower in SA compared to CL and SL (*p* < 0.001) and was lowest in SA with M amendment (Supplementary Figure S2A). SA also responded positively to BM over M addition and declined considerably over time (Supplementary Figure S2A). The Simpson index varied little with soil texture and amendment, except for a decrease over time in SA (Supplementary Figure S2A).

Community composition at the phylum and class levels clustered mainly by soil texture (PERMANOVA R^2^ = 0.625–0.669 and *p* < 0.001) (Supplementary Figure S2B). For example, abundant phyla showed distinct patterns among soil textures, with Actinomycetota (synonym Actinobacteria) declining from ~ 61% in CL to ~ 49% in SL and ~ 42% in SA and Pseudomonadota (synonym Proteobacteria) increasing from ~32% in CL to ~ 47% in SL and ~ 43% in SA (Supplementary Figure S2C). SA also contained ~10% Bacillota (synonym Firmicutes). At the lower taxonomy level (i.e., genus and species), interactions of soil texture and amendment (R^2^ = 0.531–0.541 and *p* < 0.001) and incubation time (R^2^ = 0.109–0.110 and *p* < 0.05) explained significant variations (Supplementary Figure S2B). Community separation between M and BM was obvious in SA but not in CL and SL. Yet, community variation along with the incubation time was more apparent in CL and SL than in SA.

**Figure 1:**
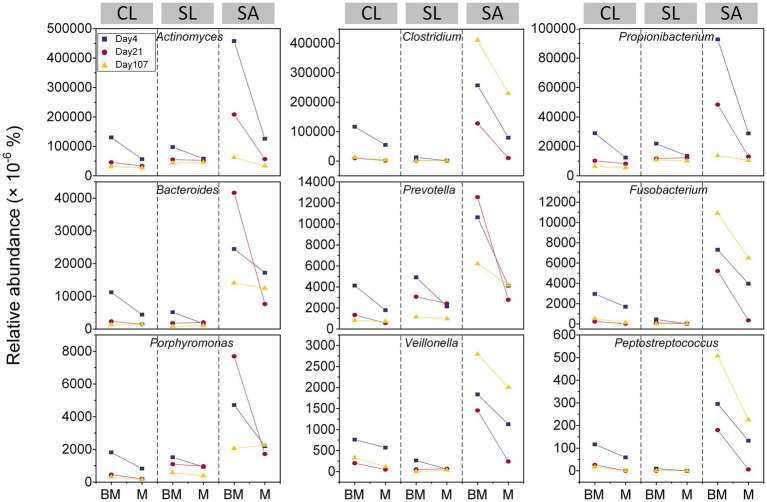
Relative abundances of facultative and obligate anaerobic bacterial genera at three time points (day 4, day 21, and day 107) during 3.5 month’s incubation following the addition of BM (biochar-manure co-compost) and M (manure compost) in clay loam (CL), silt loam (SL), and sand (SA).

Despite relatively lower abundance (i.e., < 0.5%), obligate and facultative anaerobes responded differently to amendments across soil types (i.e., CL, SL, and SA) (Figure 1). In CL, all the 9 genera peaked at the first (i.e., day 4) of the three sampling times, with BM showing greater abundances than M. In contrast, SA showed inconsistency regarding the timing when the 9 genera reached their maximal abundance and when they exhibited the greatest differences between M and BM. Among the three soils, SL generally showed the lowest abundance and minimal amendment-induced variation regardless of sampling times.

### 3.2 Metagenomic profiles of oxygen-responsive genes as affected by soil amendment

**Figure 2:**
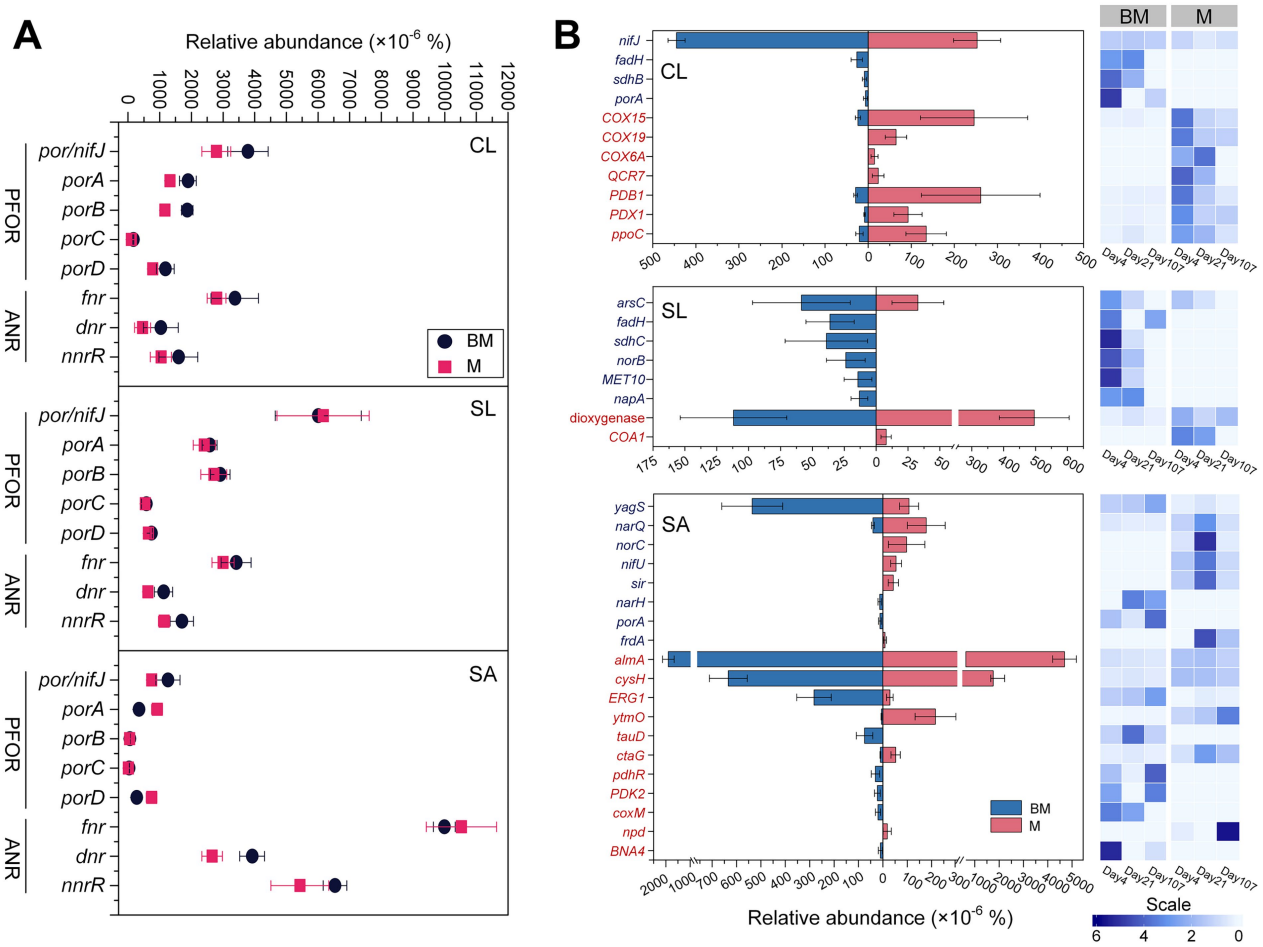
Oxygen-responsive genes based on KEGG orthology database **(A)** and eggNOG ontology database **(B)**, respectively, at three time points (day 4, day 21, and day 107) during 3.5 month’s incubation following the addition of BM (biochar-manure co-compost) and M (manure compost) in clay loam (CL), silt loam (SL), and sand (SA). Data are the means of three sampling times and standard errors for *n* = 3. Scale represents the normalized relative abundance by dividing the relative abundance by the average value across three time points. The abbreviations of functional genes shown in **(B)** are provided in Supplementary Table S4. PFOR, pyruvate:ferredoxin oxidoreductase; ANR, anaerobic regulator.

Oxygen-responsive genes were annotated with both KEGG orthology (KO) and eggNOG gene ontology (GO) databases. From the KO database, we selectively analyzed genes *pfor* (i.e., *por/nifJ*, *porA*, *porB*, *porC*, and *porD*) that encodes pyruvate:flavodoxin oxidoreductase and *anr* (i.e., *fnr*, *dnr*, and *nnrR*) that encodes transcriptional regulator for expression of anaerobic respiratory processes (Figure 2A). Pairwise comparisons showed that *anr* tended to be more abundant in BM than in M regardless of soils. However, *pfor* in response to amendments was soil-specific, inclining higher abundance in BM in CL yet no difference in SL and SA.

From the GO database, oxygen-responsive genes that were significantly different in the relative abundance between M and BM treatments (i.e., Log_2_ foldchange > 1 and *P_adj_* < 0.05) are included in Figure 2B. There were large variations among soils, meaning that a gene differing between M and BM did not appear in all three soils. Nonetheless, three soils expressed similar trends, with anaerobic process-related genes more abundant in BM and aerobic process-related genes more abundant in M (Figure 2B).

### 3.3 Metagenomic profiles of C-degradation and oxidative-stress responsive genes

**Figure 3:**
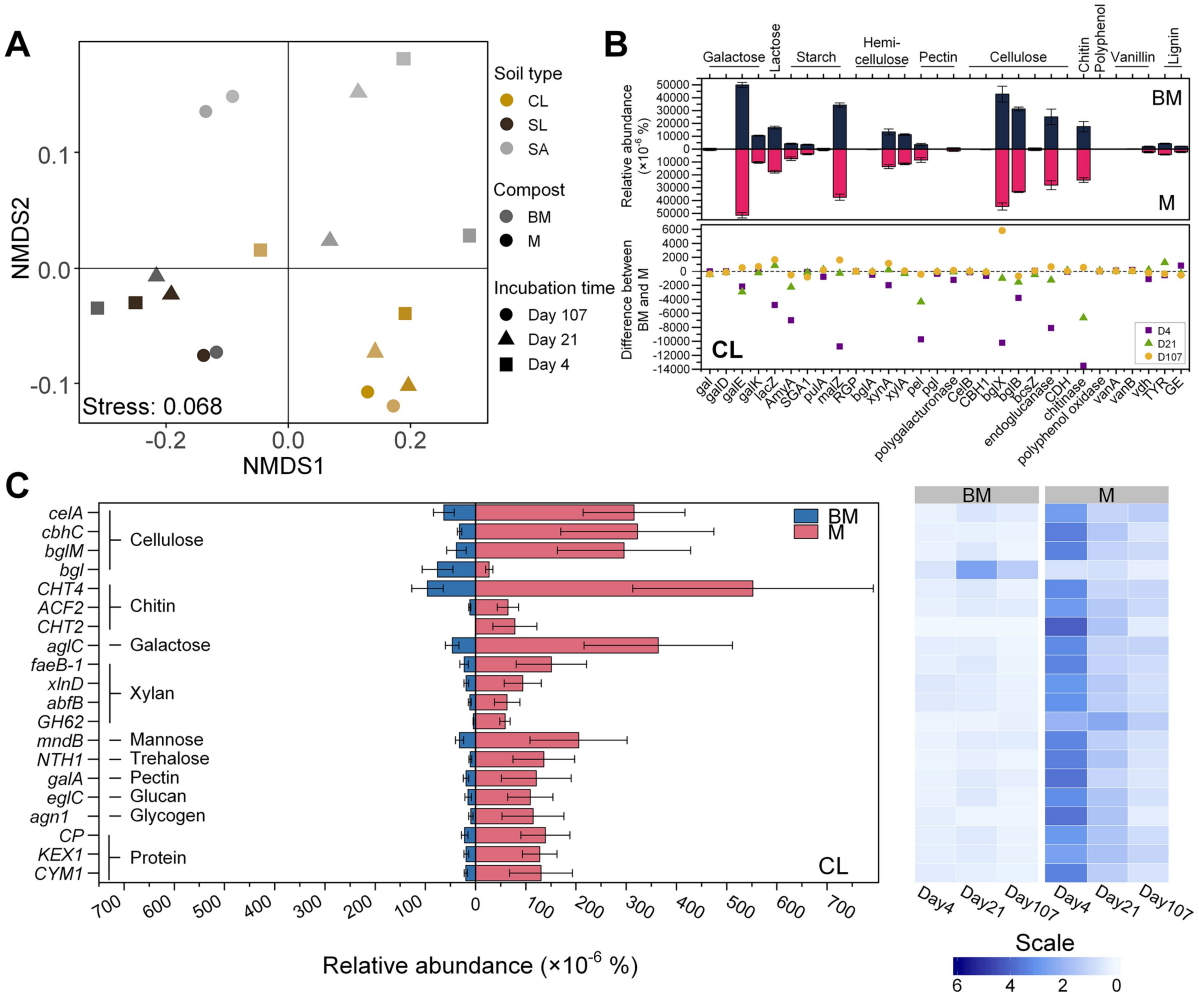
Carbon-degradation genes based on metagenomic sequencing analysis. **(A)** Non-metric multidimensional scaling (NMDS) of carbon-degradation genes based on KEGG KO number. **(B)** Relative abundances of carbon-degradation genes in clay loam (CL) based on KEGG orthology database. The bar plot shows the difference of relative abundance between BM (biochar-manure co-compost) and M (manure compost) treatment. Error bars are standard errors for *n* = 3. The scatter plot compares the difference between BM and M among three time points (day 4, day 21, and day 107) during the incubation period. **(C)** Relative abundances of carbon-degradation genes in clay loam (CL) based on eggNOG ontology database. The heatmap shows the relative abundance at three time points (day 4, day 21, and day 107) during 107 days’ incubation. Scale represents the normalized relative abundance by dividing the relative abundance by the average value across three time points.

Functional genes related to C degradation (30 genes related to the degradation of galactose, lactose, starch, hemicellulose, pectin, cellulose, chitin, polyphenol, vanillin, and lignin) were collected from the samples based on KO database (Supplementary Table S5). PERMANOVA results showed that the beta diversity of these genes was primarily affected by soil texture (*R^2^* = 0.570, *p* < 0.001), as visualized by NMDS (Figure 3A). Regardless of soil texture, however, *gal* and *galD* involved in galactose degradation, *amyA* in starch degradation, *pel* in pectin degradation, and chitinase-encoding gene were significantly affected by compost type, being more abundant in M than in BM (*p* < 0.05). The remaining genes showed soil- and incubation time-specific effects of compost type. In CL, most C-degradation genes were more abundant in M than in BM, especially at early stage of incubation (day 4). Such a variation became less pronounced by the middle stage of incubation (day 21) and nearly vanished by the end of the incubation (Figure 3B). A comparable yet less pronounced pattern was observed in SL (Supplementary Figure S3A) while the trend was less obvious in SA (Supplementary Figure S3B).

The data derived from the GO database exhibited analogous trends. Specifically, the number of C-degradation genes displaying significant differences between BM and M was greater in CL compared to SL and SA (Figure 3C; Supplementary Figures S3C,D). Furthermore, in CL, all these genes exhibited significantly reduced levels in BM when compared to M (Figure 3C), but this distinction is less apparent in SL and SA, with a few genes even showing a contrasting trend (Supplementary Figures S3C,D).

**Figure 4:**
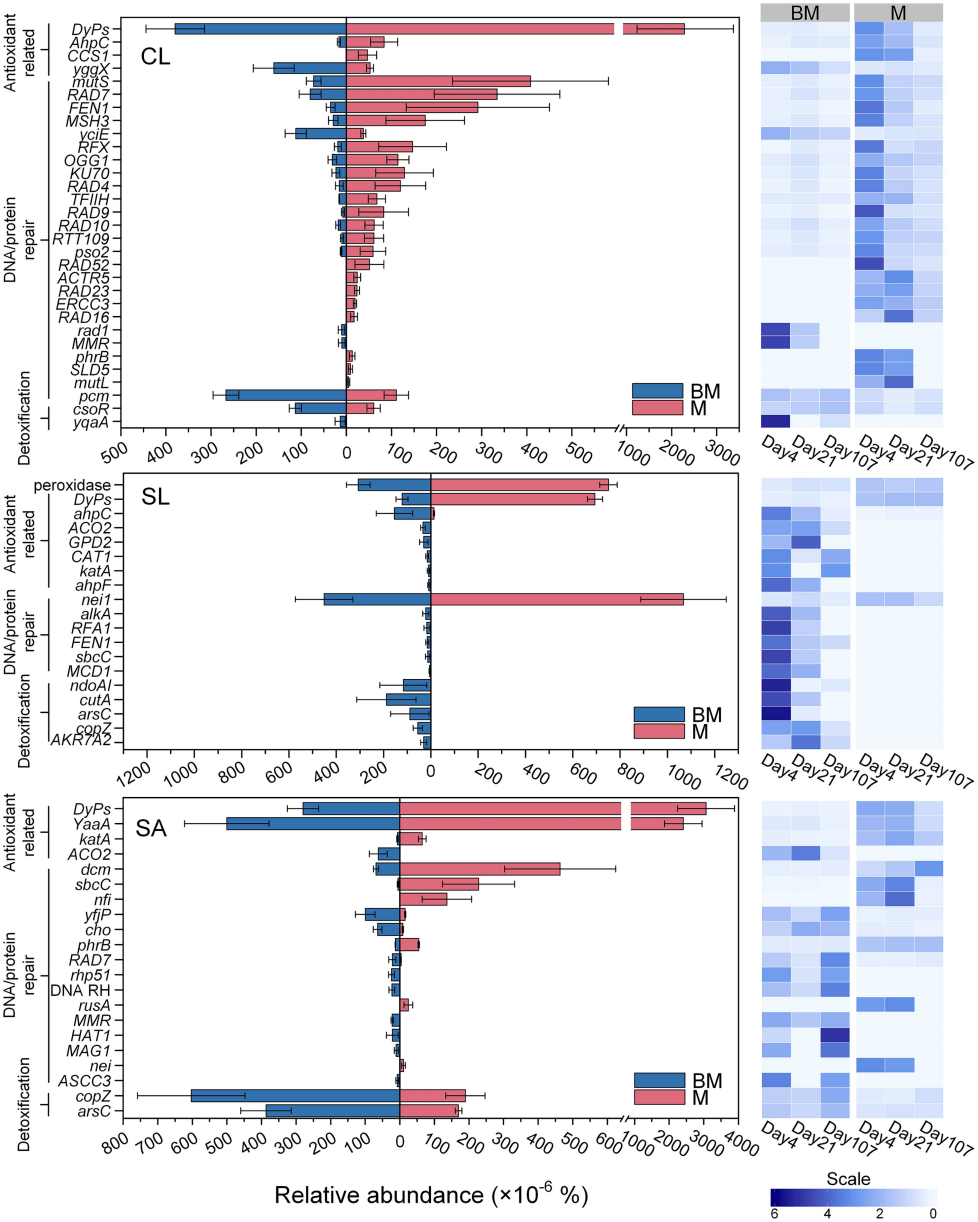
Relative abundances of oxidative-stress responsive genes based on eggNOG Ontology database in clay loam (CL), silt loam (SL), and sand (SA), respectively, following the addition of BM (biochar-manure co-compost) and M (manure compost). The bar plots compare the difference between BM and M. Error bars are standard errors for *n* = 3. The heatmaps show the relative abundance at three time points (day 4, day 21, and day 107) during 107 days’ incubation. Scale represents the normalized relative abundance by dividing the relative abundance by the average value across three time points.

As a result of organics degradation, the functional genes related to oxidative-stress response were detected from all the samples. Based on GO database, the distribution patterns of genes related to antioxidant enzymes, DNA repair mechanisms, and detoxification were distinct between BM and M and varied with soils (Figure 4). In CL, most detected antioxidant- and DNA repair-related genes were more abundant in M than in BM (*p* < 0.05), especially at early stage of incubation (day 4). This pattern was less obvious in SL and SA. However, detoxification-related genes were significantly more abundant in BM than in M regardless of soil textures (Figure 4). Based on the KO database, however, the genes related to antioxidant enzymes (e.g., superoxide dismutase, catalase, and peroxidase) were not significantly different between BM and M (Data not shown).

### 3.4 Population size of N-cycling microbes and metagenomic profiles of N-cycling genes

Populations of nitrifiers, represented by the copy number of AOA and AOB *amoA*, and denitrifiers, represented by the copy number of *nirK*, *nirS*, and *nosZ* varied significantly with soil and incubation time (Supplementary Figure S4A). Little difference in the copy numbers of all these genes was observed between BM and M. However, in SA, *nirK*-clade III at all the three time points and *nirK*-clade I on day 4 and day 21 were more abundant in M than in BM (*p* < 0.001) (Supplementary Figure S4B).

**Figure 5:**
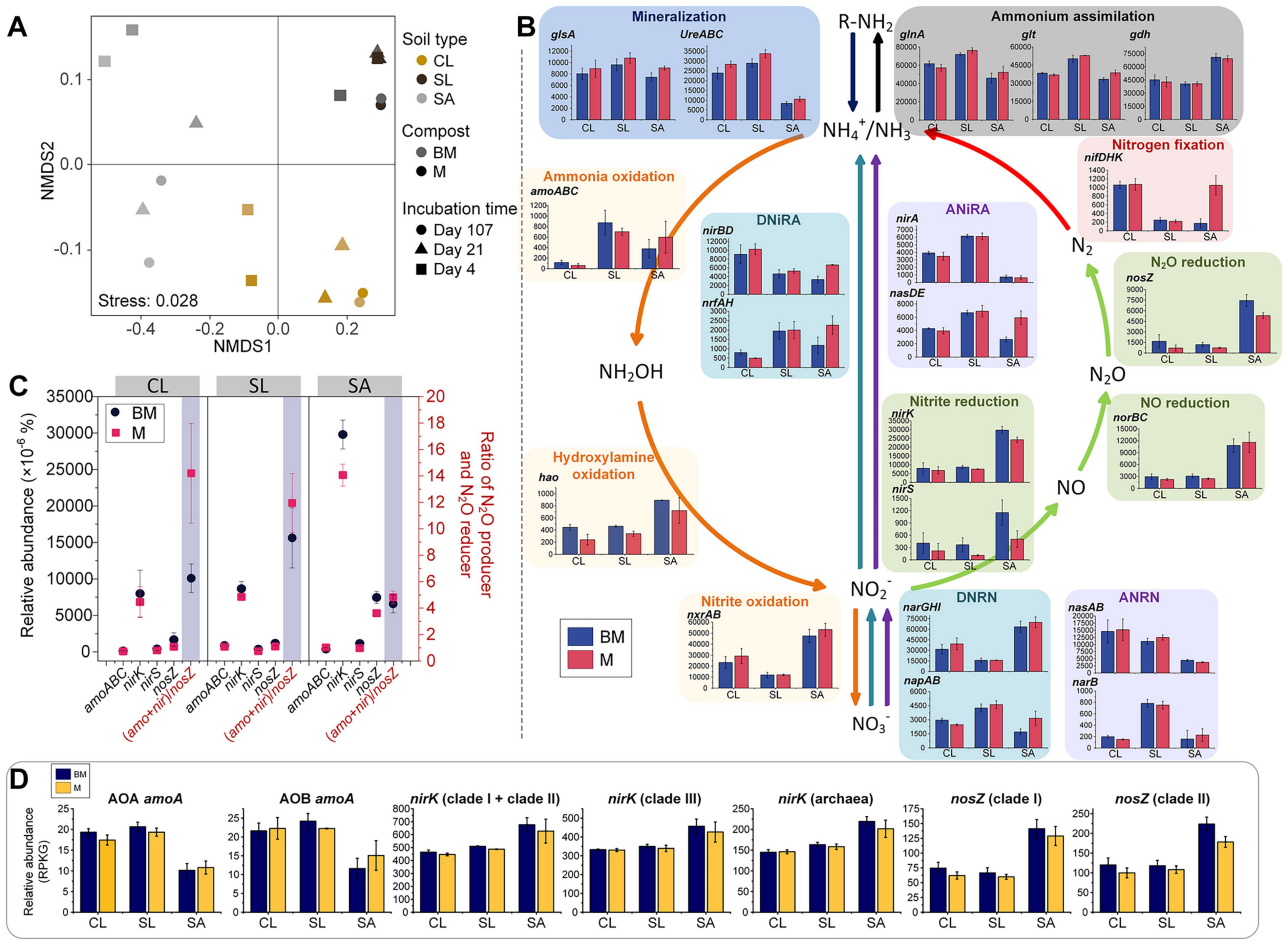
Nitrogen-cycling genes based on metagenomic sequencing analysis. **(A)** Non-metric multidimensional scaling (NMDS) of N-cycling genes based on KEGG orthology database. **(B)** Relative abundances of nitrogen-cycling genes or pathways. Different colors represent different nitrogen transformation pathways. **(C)** Relative abundances of key nitrogen-cycling functional genes (based on KEGG orthology database) related to N_2_O production and reduction as well as their ratios in different soil textures with BM or M amendment. **(D)** Relative abundances of key nitrogen-cycling functional genes (Blast result) related to N_2_O production and reduction in different soil textures with BM or M amendment. DNRN, dissimilatory nitrate reduction to nitrite; DNiRA, dissimilatory nitrite reduction to ammonium; ANRN, assimilatory nitrate reduction to nitrite; ANiRA, assimilatory nitrite reduction to ammonium; CL, clay loam; SL, silt loam; SA, sand; BM, biochar-manure co-compost; M, manure compost. RPKG, reads per kb per genome equivalent; AOA, ammonia oxidizing archaea; AOB, ammonia oxidizing bacteria. Error bars are standard errors for *n* = 3.

Forty-two functional genes involved in seven N-cycling processes (nitrification, denitrification, dissimilatory nitrate reduction to ammonia (DNRA), assimilatory nitrate reduction to ammonia (ANRA), N mineralization, ammonium assimilation, and N fixation) were selected from the KO database (Supplementary Table S6). PERMANOVA showed that the distribution pattern of microbial N metabolism functional genes was strongly influenced by soil texture (*R^2^* = 0.799, *p* < 0.001) (Figure 5A). In general, soil texture significantly influenced the relative abundances of N-cycling genes or pathways, except for *nirS* and *glsA* (*p* < 0.01). Nitrification- and denitrification-related genes/pathways were more abundant in SA or SL, while DNRA-, ANRA-, N mineralization-, ammonium assimilation-, and N fixation-related genes/pathways were more abundant in SL or CL (Figure 5B). Only a few genes/pathways were affected by compost type alone (*hao* and *ureABC*, *p* < 0.05 for both) or by compost-soil interactions (*nifDHK*, *p* < 0.01; *nasDE*, *p* < 0.05) (Figure 5B). While the relative abundance of key functional genes involved in N_2_O production (specifically *amoABC*, *nirK*, and *nirS*) and the gene responsible for N_2_O reduction (*nosZ*) were not statistically different between BM and M across soil textures, their ratios were lower in BM compared to M in CL and SL (*p* < 0.1 and *p* < 0.01, respectively) (Figure 5C). The relative abundance of *amoA*, *nirK* or *nosZ* derived from the BLAST pipeline also differed mainly among soils textures (*p* < 0.01 for all), being lower in SA than in CL and SL for *amoA*, but opposite for *nirK* and *nosZ* (Figure 5D). Compost type and interaction with soil texture did not show a significant effect. However, compost type exhibited a marginally significant effect on *nosZ* clade II (*p* = 0.054), with the relative abundance in SA being higher in BM than in M treatment (*p* < 0.05).

## 4 Discussion

Compared to M, BM has been found to reduce CO_2_ and N_2_O emissions; yet the degree in reduction is soil-texture dependent (Gao et al., 2023; Yuan et al., 2017). The underlying mechanism has been linked to biochar-modulations in soil structure and thus soil aeration (Hu et al., 2024). Our study provided multiple lines of evidence that microbiome traits effectively reflect pore-scale environments that could not be reliably inferred by bulk soil properties. While soil texture and compost/biochar characteristics undoubtedly influence microbial communities, our objective here was not to disentangle these individual effects but to determine whether microbial traits can serve as reliable indicators of pore-scale oxygen status across different soil-amendments.

### 4.1 Bacterial community diversity and compositional traits of soil aeration status

Biochar in BM increased soil microporosity, leading to slower drainage and reduced air diffusion, thereby raising the likelihood of hypoxia (Gentry et al., 2021). Consequently, we expected a higher abundance of obligate anaerobic bacteria in BM than in M. Unlike obligate anaerobes that lack defense mechanisms against reactive oxygen species (ROS) (Lu and Imlay, 2021), facultative anaerobes can tolerate oxygen but thrive better under anaerobic conditions (Bowden, 1996). Our findings showed that both obligate and facultative anaerobic bacteria were more abundant in BM than in M across soil textures, though still relatively low (< 0.5%). This suggested that BM created localized hypoxic or anoxic conditions more effectively than M.

The compositional trait of the soil microbiome seemed reliable for indicating soil aeration status. Previous analyses of soil properties and enzyme activities suggested that SL was more aerated than CL and SA (Hu et al., 2024). This aligned with the interaction between organic amendments and soil textures affecting relative abundance of anaerobes. The least difference in SL implied that microporosity variations between M and BM were insufficient to generate discernable differences in soil aeration when soil structure could provide better aeration. This was further supported by the lack of microbial diversity differences between BM and M in SL, unlike in CL and SA. In SL, the significant change in pore size distribution would likely impact microbial diversity by influencing trophic interactions and the resource heterogeneity (Xia et al., 2020). In contrast, our data from CL and SA aligned with Hartmann et al. (2014), showing that a significant increase in the proportion of small pores would promote the proliferation of anaerobes (Hartmann et al., 2014).

It was unsurprising that microbial composition varied across soils, since they came from different field sites and environmental conditions. However, we were surprised by the sensitivity of anaerobes to pore-scale aeration changes in SA. In fine-textured CL, anaerobic conditions emerged rapidly after the addition of biochar-manure co-composts. In contrast, coarse-textured SA exhibited different outburst patterns, with some anaerobes being more abundant at the end of incubation. This may be due to persistent anaerobic conditions in small pores, where slow or limited oxygen diffusion prevented recovery (Li et al., 2024b).

### 4.2 Gene evidence of soil aeration status

Anaerobic metabolism is another key indicator of soil aeration. We demonstrated that soil aeration status, inferred from CO_2_ and N_2_O emissions, was paralleled with changes in the relative abundance of genes for anaerobic metabolism (Hu et al., 2024). Under anoxic conditions, microbes acquire energy via fermentation or anaerobic respiration. While denitrification and fermentation can occur simultaneously in soils with organic amendments, fermentation often requires more severe anaerobic conditions (Paul and Beauchamp, 1989; Pidello et al., 1996; Tiedje et al., 1984). Compared to fine-textured soils, SA was assumed to have fewer small pores and weaker anaerobic conditions. This supposition appeared to align well with the lower relative abundance of fermentation-related genes in SA than in other soils. Our data also indicated that BM increased the proportion of small pores, particularly in fine-textured CL, enhancing fermentation. Conversely, genes involved in O_2_/redox sensing transcription regulators, especially *dnr* and *nnrR* for denitrification, were more abundant in SA than in SL and CL, supporting the idea that denitrification was preferred over fermentation under milder anaerobic conditions (Tiedje et al., 1984). Although anaerobic pores existed in all three soils, our data suggested that BM addition promoted more hypoxic conditions in coarse-textured soils than in fine-textured soils.

Dissimilatory nitrate reduction to ammonium (DNRA) occurs under stricter anaerobic conditions than denitrification and fermentation (Tiedje et al., 1984). NADH-dependent nitrite reductase *nirBD* was significantly more abundant in CL than in SL and SA, suggesting DNRA-favorable pores were more prevalent in CL. Although periplasmic cytochrome nitrate reductase *nrfAH* showed an opposite trend, being lower in CL than SL and SA, its relative abundance was substantially lower than the relative abundance of *nirBD*, perhaps implying that *nirBD*-containing bacteria dominated DNRA under our experimental conditions.

Based on GO database, different sets of reductive/oxidative genes were identified from three soils, with significant differences between BM and M. Still, fermentation-related genes were more abundant in CL than in other soils and in BM than in M. This augmented the findings from the compositional trait of the microbiome and the KO database-derived anaerobic metabolism, highlighting that in fine-textured CL, pores favoring fermentation might surpass pores favoring denitrification.

### 4.3 Development of anaerobic soil pores following organic amendments

In aerobic soils, temporary anaerobic conditions can be developed in pores where microbial respiration depletes oxygen. The intensity of microbial respiration can be perceived from the activation of antioxidant defenses and repair systems to scavenge aerobic respiration-induced ROS (Seixas et al., 2021). Greater aerobic respiration leads to increased ROS production, triggering more abundant antioxidant defenses and DNA/protein repair genes. Across soil textures, M-amended soils exhibited higher abundances of these genes than BM-amended soils, suggesting stronger aerobic respiration and thus higher CO_2_ emissions. This was corroborated with the relative abundance of genes involved in organic compound degradation. Sharp declines in the abundance of those genes over time, particularly in M-amended soils, likely resulted from the reduced availability of readily-degradable organic compounds and/or the cascading effect of microbial respiration-induced oxygen shortage slowing further degradation. Hypoxic/anoxic hotspots are expected in organic-rich microsites with limited oxygen diffusion (Borer et al., 2018). Among M-amended soils, fine-textured CL was most likely to face diffusion limitations due to its higher microporosity, leading to more oxygen-limited small pores and a more pronounced decline in organic decomposition genes compared to SL and SA.

Lower abundance of genes for carbon degradation in BM-amended soils validated that biochar promoted carbon stabilization during composting due to its high adsorption capacity, microporosity, and basic functional groups (Dissanayake et al., 2020; Oladele, 2019; Wang et al., 2023a). It often manifests at the soil scale as the suppression of soil enzyme activities (Feng et al., 2023; Foster et al., 2018). Unlike M-amended soils where carbon overflow might promote ROS and subsequently trigger DNA damage repair (Thomas et al., 2014), microbes in BM-amended soils appeared not to deal with much oxidative stress or DNA repair, since involved genes with high CPM (e.g., *DyPs*) were less abundant. Besides lower organic carbon availability, more anaerobiosis in BM-amended soils might offer an alternative explanation to reduced C degradation. The presence of anaerobic pores in ‘apparent’ aerobic soils can considerably shift C cycling to less efficient anaerobic metabolism, leading to lower CO_2_ emissions (Keiluweit et al., 2017). Genes with low CPM (~ 1–2 CPM on average) were more abundant in BM than M in SL and SA but not in CL, suggesting greater heterogeneity in pore size and associated resource distribution. It also suggested that biochar in BM exerted more impact in SA than in fine-textured soils. Similar soil-dependent effects of biochar on aeration were reported by Wang et al. (2023b).

Notably, genes related to detoxification, specifically those involved in Cu and As homeostasis and/or resistance, were more abundant in BM than in M-amended soils, indicating that biochar addition increased metal concentrations in composts. Biochar produced from treated or milled pine material contain certain levels of heavy metals due to the use of chromated copper arsenate, a common wood preservative (Askeland et al., 2019). The detected differences in these gene abundances between M and BM treatments suggested the high sensitivity of metagenomics as a tool to monitor environmental changes within the soil matrix.

### 4.4 Sensitivity of N-cycling genes in predicting N_2_O emissions

qPCR is widely used to assess soil N transformations (Hu et al., 2021), despite limitations such as primer coverage and bias (Smith and Osborn, 2009). Our qPCR results aligned with soil properties and processes. AOA populations kept low in ammonium-dominant CL and reduced with decreasing ammonium in SA (Hu et al., 2024), consistent with the preference of AOA for low ammonium concentrations over AOB (Di et al., 2009; Lin et al., 2021; Martens-Habbena et al., 2009). In contrast, AOB proliferated rapidly in SL and SA but much more slowly in CL, likely due to poor aeration from CL’s higher microporosity and rapid oxygen depletion by microbial respiration. As decomposition proceeded and bioavailable organics decreased, AOB growth accelerated in CL, suggesting gradual amelioration of anaerobic stress due to lower oxygen consumption through microbial respiration. However, the abundance of denitrification-related genes (*nirK*, *nirS*, and *nosZ*) did not consistently reflect BM’s N_2_O mitigation effects, as gene copy numbers were not consistently lower in BM than M across three soils. This may be due to qPCR primers targeting only subsets of denitrifiers, limiting sensitivity to treatment effects. To address this, we used multiple primer sets targeting *nirK* clades, including *α*-, *β*- and *γ*-Proteobacteria in clade I; Proteobacteria, Actinobacteria, Bacteroidetes, Firmicutes, Archaea, and other taxa in clade II; and Actinobacteria in clade III (Luo et al., 2021; Wei et al., 2015). Yet, these clade-specific gene copy numbers also failed to conclusively support the lower N_2_O emission in BM than in M across different soil textures. Notably, denitrifier abundance in CL was high (~ 1 × 10^8^ copies of *nirK* and *nosZ)*, yet N_2_O-emissions were ~ 100-fold lower than in SA, suggesting a large proportion of denitrifiers were inactive. This may explain the unexpectedly lower sensitivity of qPCR in detecting soil management effects in our study.

We then explored whether shotgun metagenomics would better link microbiome data to N_2_O emissions. The BLAST-based annotation approach did not establish a clear correlation between gene copy numbers and N_2_O emissions. However, *de novo* strategy could, to some degree, reflect N transformations at the soil scale. The ratio of genes for N_2_O production and consumption, i.e., (*amoA*+*nirK*+*nirS*)/*nosZ* was statistically lower in BM-amended soils compared to M-amended soils, suggesting BM’s greater potential for mitigating N_2_O emissions. Additionally, SA had the highest relative abundance of denitrification genes, aligning with its highest observed N_2_O emissions.

This study has several limitations that should be acknowledged. Pore-scale oxygen availability was inferred indirectly from microbial traits rather than measured directly, which introduces some uncertainty. The soils originated from different sites, so part of the microbial variation may reflect site-specific history beyond texture differences. Functional gene profiles derived from metagenomics and qPCR also have inherent biases, and not all gene abundances aligned with observed N_2_O fluxes. Finally, the work was conducted under controlled incubation conditions, which may not fully capture the complexity of dynamic field environments.

Despite these limitations, our findings highlight the cascading effect of organic degradation on soil aeration and N_2_O emissions. It exemplifies how microbial respiration interacts with soil structure, influencing hypoxic and anoxic porosity. Metagenomic traits provide a sensitive tool for detecting pore-scale environmental shifts, improving our mechanistic understanding of soil-dependent GHG emissions following organic amendments. Further research is needed to identify robust indicator genes for predicting soil processes at the pore scale.

## Data Availability

The datasets presented in this study can be found in online repositories. The names of the repository/repositories and accession number(s) can be found in the article/Supplementary material.
