# Supplementary material for: Metagenomic evidence clarifies the texture-dependent cascading effects of organic degradation on soil hypoxia and N2O emission
Source: Front Microbiol. 2025 Sep 22;16:1670657. doi: 10.3389/fmicb.2025.1670657 (PMC12497740; doi:10.3389/fmicb.2025.1670657)
Supplement: Supplementary file 1 [file Data_Sheet_1.docx]

Supplementary data

**1. Supplementary Tables**

Table S1. Summary statistics of Illumina reads across 18 samples (i.e., 3 soil textures × 2 organic amendments × 3 sampling times)

|  | Number of raw reads | Number of clean reads |
| --- | --- | --- |
| Mean | 83,232,001 | 82,655,478 |
| Min | 59,462,904 | 59,098,544 |
| Max | 148,358,982 | 147,391,640 |
| CV (%) | 32 | 32 |

Table S2. Primers and PCR conditions used for qPCR.

| Gene | Primers | Sequence (5'-3') | Reaction parameters |
| --- | --- | --- | --- |
| *amoA*  (AOA^†^) | Arch-amoAF  Arch-amoAR | STAATGGTCTGGCTTAGACG  GCGGCCATCCATCTGTATGT | 95 ℃ for 10 min × 1 cycle; (94 ℃ for 45 s, 53 ℃ for 1 min, 72 ℃ for 1 min) × 40 cycles; 72 ℃ for 10 min × 1 cycle |
| *amoA* (AOB^†^) | *amoA* 1F  *amoA* 2R | GGGGTTTCTACTGGTGGT  CCCCTCKGSAAAGCCTTCTTC | 95 ℃ for 10 min × 1 cycle; (94 ℃ for 1 min, 56 ℃ for 45 s, 72 ℃ for 1 min) × 40 cycles; 72 ℃ for 10 min × 1 cycle |
| *nirS* | cd3aF  R3cd | GTSAACGTSAAGGARACSGG  GASTTCGGRTGSGTCTTGA | 95 ℃ for 10 min × 1 cycle; (94 ℃ for 30 s, 57 ℃ for 1 min, 72 ℃ for 1 min) × 40 cycles; 72 ℃ for 10 min × 1 cycle |
| *nirK* | F1aCu  R3Cu | ATCATGGTSCTGCCGCG  GCCTCGATCAGRTTGTGGTT | 95 ℃ for 10 min × 1 cycle; (94 ℃ for 30 s, 58 ℃ for 1 min, 72 ℃ for 1 min) × 40 cycles; 72 ℃ for 10 min × 1 cycle |
| *nosZ* | *nosZ*-I F  *nosZ*-I R | CGCRACGGCAASAAGGTSMSSGT  CAKRTGCAKSGCRTGGCAGAA | 95 ℃ for 10 min × 1 cycle; (94 ℃ for 40 s, 60 ℃ for 40 s, 72 ℃ for 1 min) × 40 cycles; 72 ℃ for 10 min × 1 cycle |
| *nirK*  (cladeI) | nirKC1F  nirKC1R | ATGGCGCCATCATGGTNYTNCC  TCGAAGGCCTCGATNARRTTRTG | 95 ℃ for 10 min × 1 cycle; (94 ℃ for 30 s, 54 ℃ for 30 s, 72 ℃ for 1 min) × 40 cycles; 72 ℃ for 10 min × 1 cycle |
| *nirK*  (cladeII) | nirKC2F  nirKC2R | TGCACATCGCCAACGGNATGTWYGG  GGCGCGGAAGATGSHRTGRTCNAC | 95 ℃ for 10 min × 1 cycle; (94 ℃ for 30 s, 56 ℃ for 30 s, 72 ℃ for 1 min) × 40 cycles; 72 ℃ for 10 min × 1 cycle |
| *nirK*  (cladeIII) | nirKC3F  nirKC3R | CATCGGCAACGGCATGYAYGGNGC  CGACCATGGCCGTGGSWNACRAANGG | 95 ℃ for 10 min × 1 cycle; (94 ℃ for 30 s, 58 ℃ for 30 s, 72 ℃ for 1 min) × 40 cycles; 72 ℃ for 10 min × 1 cycle |

^†^AOA, ammonia-oxidizing archaea; AOB, ammonia-oxidizing bacteria.

Table S3. Summary statistics of clean reads across 18 samples (i.e., 3 soil textures × 2 organic amendments × 3 sampling times) that could be classified into microbial superkingdoms by Kraken2.

|  | % of classified reads | % of superkingdom within classified reads | | | |
| --- | --- | --- | --- | --- | --- |
|  |  | Bacteria | Eukaryota | Archaea | Viruses |
| Min | 16.8 | 97.1 | 0.2 | 0.1 | 0.00 |
| Max | 35.9 | 99.9 | 2.7 | 0.4 | 0.02 |
| Mean | 25.2 | 99.0 | 0.8 | 0.2 | 0.00 |
| CV (%) | 19.9 | 0.6 | 83.0 | 40.1 | 138.5 |

Table S4. Abbreviations of functional genes given in Fig. 2B.

| Abbreviation | oxidation status | Full name |
| --- | --- | --- |
| CL |  |  |
| nifJ | Anaerobic | Pyruvate flavodoxin oxidoreductase |
| fadH | Anaerobic | Pentaerythritol trinitrate reductase activity |
| sdhB | Anaerobic | Fumarate reductase |
| porA | Anaerobic | Pyruvate ferredoxin oxidoreductase |
| COX15 | Aerobic | Cytochrome C oxidase assembly protein cox15 |
| COX19 | Aerobic | Cytochrome c oxidase assembly protein Cox19 |
| COX6A | Aerobic | Cytochrome c oxidase subunit VIa |
| QCR7 | Aerobic | Component of the ubiquinol-cytochrome c reductase complex (complex III or cytochrome b-c1 complex), which is part of the mitochondrial respiratory chain |
| PDB1 | Aerobic | The pyruvate dehydrogenase complex catalyzes the overall conversion of pyruvate to acetyl-CoA and CO2. |
| PDX1 | Aerobic | Pyruvate dehydrogenase complex component Pdx1 |
| ppoC | Aerobic | fatty acid oxygenase |
| SL |  |  |
| arsC | Anaerobic | Arsenate reductase and related |
| fadH | Anaerobic | pentaerythritol trinitrate reductase activity |
| sdhC | Anaerobic | COG2009 Succinate dehydrogenase fumarate reductase, cytochrome b subunit |
| norB | Anaerobic | Nitric oxide reductase large subunit |
| MET10 | Anaerobic | Pyruvate ferredoxin/flavodoxin oxidoreductase |
| napA | Anaerobic | nitrate reductase (NAP). Only expressed at high levels during aerobic growth. NapAB complex receives electrons from the membrane-anchored tetraheme protein NapC |
| dioxygenase | Aerobic | dioxygenase activity |
| COA1 | Aerobic | Cytochrome oxidase complex assembly protein 1 |
| SA |  |  |
| yagS | Anaerobic | CO dehydrogenase flavoprotein C-terminal domain |
| narQ | Anaerobic | COG3850 Signal transduction histidine kinase, nitrate nitrite-specific |
| norC | Anaerobic | Nitric-oxide reductase subunit C |
| nifU | Anaerobic | Nitrogen fixation protein NifU |
| sir | Anaerobic | nitrite sulfite reductase hemoprotein beta-component ferrodoxin domain protein |
| narH | Anaerobic | one of 3 nitrate reductases in E. coli and in E. coli is expressed when nitrate levels are high |
| porA | Anaerobic | pyruvate flavodoxin ferredoxin oxidoreductase |
| frdA | Anaerobic | fumarate reductase, flavoprotein subunit |
| almA | Aerobic | Flavin-binding monooxygenase-like |
| cysH | Aerobic | Phosphoadenosine phosphosulfate reductase family |
| ERG1 | Aerobic | Squalene monooxygenase |
| ytmO | Aerobic | Alkane 1-monooxygenase |
| tauD | Aerobic | taurine dioxygenase |
| ctaG | Aerobic | Cytochrome c oxidase assembly protein CtaG/Cox11 |
| pdhR | Aerobic | pyruvate dehydrogenase complex |
| PDK2 | Aerobic | Pyruvate dehydrogenase kinase |
| coxM | Aerobic | cytochrome c oxidase subunit II |
| npd | Aerobic | nitronate monooxygenase activity |
| BNA4 | Aerobic | kynurenine 3-monooxygenase activity |

Table S5. Carbon degradation-related genes and their KEGG KO number.

| **Carbon type** | **KO** | **Definition** |
| --- | --- | --- |
| Calactose | K00035 | gal; D-galactose 1-dehydrogenase [EC:1.1.1.48] |
|  | K22215 | galD; galactose dehydrogenase [EC:1.1.1.48 1.1.1.120] |
|  | K01784 | galE, GALE; UDP-glucose 4-epimerase [EC:5.1.3.2] |
|  | K00849 | galK; galactokinase [EC:2.7.1.6] |
| Lactose | K01190 | lacZ; beta-galactosidase [EC:3.2.1.23] |
| Starch | K01176 | AMY, amyA, malS; alpha-amylase [EC:3.2.1.1] |
|  | K01178 | SGA1; glucoamylase [EC:3.2.1.3] |
|  | K01200 | pulA; pullulanase [EC:3.2.1.41] |
|  | K01187 | malZ; alpha-glucosidase [EC:3.2.1.20] |
| Hemicellulose | K13379 | RGP, UTM; reversibly glycosylated polypeptide / UDP-arabinopyranose mutase [EC:2.4.1.- 5.4.99.30] |
|  | K01188 | E3.2.1.21; beta-glucosidase [EC:3.2.1.21] |
|  | K01181 | E3.2.1.8, xynA; endo-1,4-beta-xylanase [EC:3.2.1.8] |
|  | K01805 | xylA; xylose isomerase [EC:5.3.1.5] |
| Pectin | K01728 | pel; pectate lyase [EC:4.2.2.2] |
|  | K01732 | E4.2.2.10; pectin lyase [EC:4.2.2.10] |
|  | K01184 | E3.2.1.15; polygalacturonase [EC:3.2.1.15] |
| Cellulose | K19357 | CELB; cellulase [EC:3.2.1.4] |
|  | K01225 | CBH1; cellulose 1,4-beta-cellobiosidase [EC:3.2.1.91] |
|  | K05349 | bglX; beta-glucosidase [EC:3.2.1.21] |
|  | K05350 | bglB; beta-glucosidase [EC:3.2.1.21] |
|  | K20542 | bcsZ; endoglucanase [EC:3.2.1.4] |
|  | K01179 | E3.2.1.4; endoglucanase [EC:3.2.1.4] |
|  | K19069 | CDH; cellobiose dehydrogenase (acceptor) [EC:1.1.99.18] |
| Chitin | K01183 | E3.2.1.14; chitinase [EC:3.2.1.14] |
| Polyphenol | K00422 | E1.10.3.1; polyphenol oxidase [EC:1.10.3.1] |
| Vanillin | K03862 | vanA; vanillate monooxygenase [EC:1.14.13.82] |
|  | K03863 | vanB; vanillate monooxygenase ferredoxin subunit |
|  | K21802 | vdh; vanillin dehydrogenase [EC:1.2.1.67] |
| Lignin | K00505 | TYR; tyrosinase [EC:1.14.18.1] |
|  | K25010 | (4-O-methyl)-D-glucuronate---lignin esterase [EC:3.1.1.117] |

Table S6. Nitrogen metabolism-related pathways, genes, and KEGG KO number.

| **Pathway** | **Sub-pathway** | **Gene** | **KO** | **Definition** |
| --- | --- | --- | --- | --- |
| Nitrogen fixation | Nitrogen fixation | *nifDHK* | K02586 | nifD; nitrogenase molybdenum-iron protein alpha chain [EC:1.18.6.1] |
|  |  |  | K02588 | nifH; nitrogenase iron protein NifH [EC:1.18.6.1] |
|  |  |  | K02591 | nifK; nitrogenase molybdenum-iron protein beta chain [EC:1.18.6.1] |
| Nitrification | Ammonia oxidation | *amoABC* | K10944 | pmoA-amoA; methane/ammonia monooxygenase subunit A [EC:1.14.18.3 1.14.99.39] |
|  |  |  | K10945 | pmoB-amoB；methane/ammonia monooxygenase subunit B |
|  |  |  | K10946 | pmoC-amoC; methane/ammonia monooxygenase subunit C |
|  | hydroxylamine oxidation | *hao* | K10535 | hao;hydroxylamine dehydrogenase [EC:1.7.2.6] |
|  | Nitrite oxidation | *nxrAB* | K00370 | narG, narZ, nxrA; nitrate reductase / nitrite oxidoreductase, alpha subunit [EC:1.7.5.1 1.7.99.4] |
|  |  |  | K00371 | narH, narY, nxrB; nitrate reductase / nitrite oxidoreductase, beta subunit [EC:1.7.5.1 1.7.99.4] |
| Denitrification | Nitrate reduction | *narGHI* | K00370 | narG, narZ, nxrA; nitrate reductase / nitrite oxidoreductase, alpha subunit [EC:1.7.5.1 1.7.99.4] |
|  |  |  | K00371 | narH, narY, nxrB; nitrate reductase / nitrite oxidoreductase, beta subunit [EC:1.7.5.1 1.7.99.4] |
|  |  |  | K00374 | narI, narV; nitrate reductase gamma subunit [EC:1.7.5.1 1.7.99.4] |
|  |  | *napAB* | K02567 | napA; periplasmic nitrate reductase NapA [EC:1.7.99.4] |
|  |  |  | K02568 | napB; cytochrome c-type protein NapB |
|  | Nitrite reduction | *nirK* | K00368 | nirK; nitrite reductase (NO-forming) [EC:1.7.2.1] |
|  |  | *nirS* | K15864 | nirS; nitrite reductase (NO-forming) / hydroxylamine reductase [EC:1.7.2.1 1.7.99.1] |
|  | Nitric oxide reduction | *norBC* | K04561 | norB; nitric oxide reductase subunit B [EC:1.7.2.5] |
|  |  |  | K02305 | norC; nitric oxide reductase subunit C |
|  | Nitrous oxide reduction | *nosZ* | K00376 | nosZ; nitrous-oxide reductase [EC:1.7.2.4] |
| DNRA | DNRN | *narGHI* | K00370 | narG, narZ, nxrA; nitrate reductase / nitrite oxidoreductase, alpha subunit [EC:1.7.5.1 1.7.99.4] |
|  |  |  | K00371 | narH, narY, nxrB; nitrate reductase / nitrite oxidoreductase, beta subunit [EC:1.7.5.1 1.7.99.4] |
|  |  |  | K00374 | narI, narV; nitrate reductase gamma subunit [EC:1.7.5.1 1.7.99.4] |
|  |  | *napAB* | K02567 | napA; periplasmic nitrate reductase NapA [EC:1.7.99.4] |
|  |  |  | K02568 | napB; cytochrome c-type protein NapB |
|  | DNiRA | *nirBD* | K00362 | nirB; nitrite reductase (NADH) large subunit [EC:1.7.1.15] |
|  |  |  | K00363 | nirD; nitrite reductase (NADH) small subunit [EC:1.7.1.15] |
|  |  | *nrfAH* | K03385 | nrfA; nitrite reductase (cytochrome c-552) [EC:1.7.2.2] |
|  |  |  | K15876 | nrfH; cytochrome c nitrite reductase small subunit |
| ANRA | ANRN | *nasAB* | K00372 | nasA; assimilatory nitrate reductase catalytic subunit [EC:1.7.99.4] |
|  |  |  | K00360 | nasB; assimilatory nitrate reductase electron transfer subunit [EC:1.7.99.4] |
|  |  | *narB* | K00367 | narB; ferredoxin-nitrate reductase [EC:1.7.7.2] |
|  | ANiRA | *nirA* | K00366 | nirA; ferredoxin-nitrite reductase [EC:1.7.7.1] |
|  |  | *nasDE* | K26139 | nasD; nitrite reductase [NAD(P)H] large subunit [EC:1.7.1.4] |
|  |  |  | K26138 | nasE; nitrite reductase [NAD(P)H] small subunit [EC:1.7.1.4] |
| Ammonium assimilation | GS/GOGAT | *glnA* | K01915 | glnA, GLUL; glutamine synthetase [EC:6.3.1.2] |
|  |  | *glt* | K00264 | GLT1; glutamate synthase (NADPH/NADH) [EC:1.4.1.13 1.4.1.14] |
|  |  |  | K00265 | gltB; glutamate synthase (NADPH/NADH) large chain [EC:1.4.1.13 1.4.1.14] |
|  |  |  | K00266 | gltD; glutamate synthase (NADPH/NADH) small chain [EC:1.4.1.13 1.4.1.14] |
|  |  |  | K00284 | E1.4.7.1; glutamate synthase (ferredoxin) [EC:1.4.7.1] |
|  | gdh | *gdh* | K00260 | gudB, rocG; glutamate dehydrogenase [EC:1.4.1.2] |
|  |  |  | K15371 | GDH2; glutamate dehydrogenase [EC:1.4.1.2] |
|  |  |  | K00261 | GLUD1_2, gdhA; glutamate dehydrogenase (NAD(P)+) [EC:1.4.1.3] |
|  |  |  | K00262 | E1.4.1.4, gdhA; glutamate dehydrogenase (NADP+) [EC:1.4.1.4] |
| Mineralization | urease | *ure* | K01427 | URE; urease [EC:3.5.1.5] |
|  |  |  | K01428 | ureC; urease subunit alpha [EC:3.5.1.5] |
|  |  |  | K01429 | ureB; urease subunit beta [EC:3.5.1.5] |
|  |  |  | K01430 | ureA; urease subunit gamma [EC:3.5.1.5] |
|  |  |  | K14048 | ureAB; urease subunit gamma/beta [EC:3.5.1.5] |
|  | glutaminase | *gls* | K01425 | glsA, GLS; glutaminase [EC:3.5.1.2] |

**2. Supplementary Figures**

Fig. S1. Cumulative CO_2_ and N_2_O emissions from soils of three textural classes subjected to two amendments (M and BM) on day 4, 21and 107 of the incubation. M and BM represent manure compost and biochar-manure compost, respectively. Error bars are standard errors for n = 3.


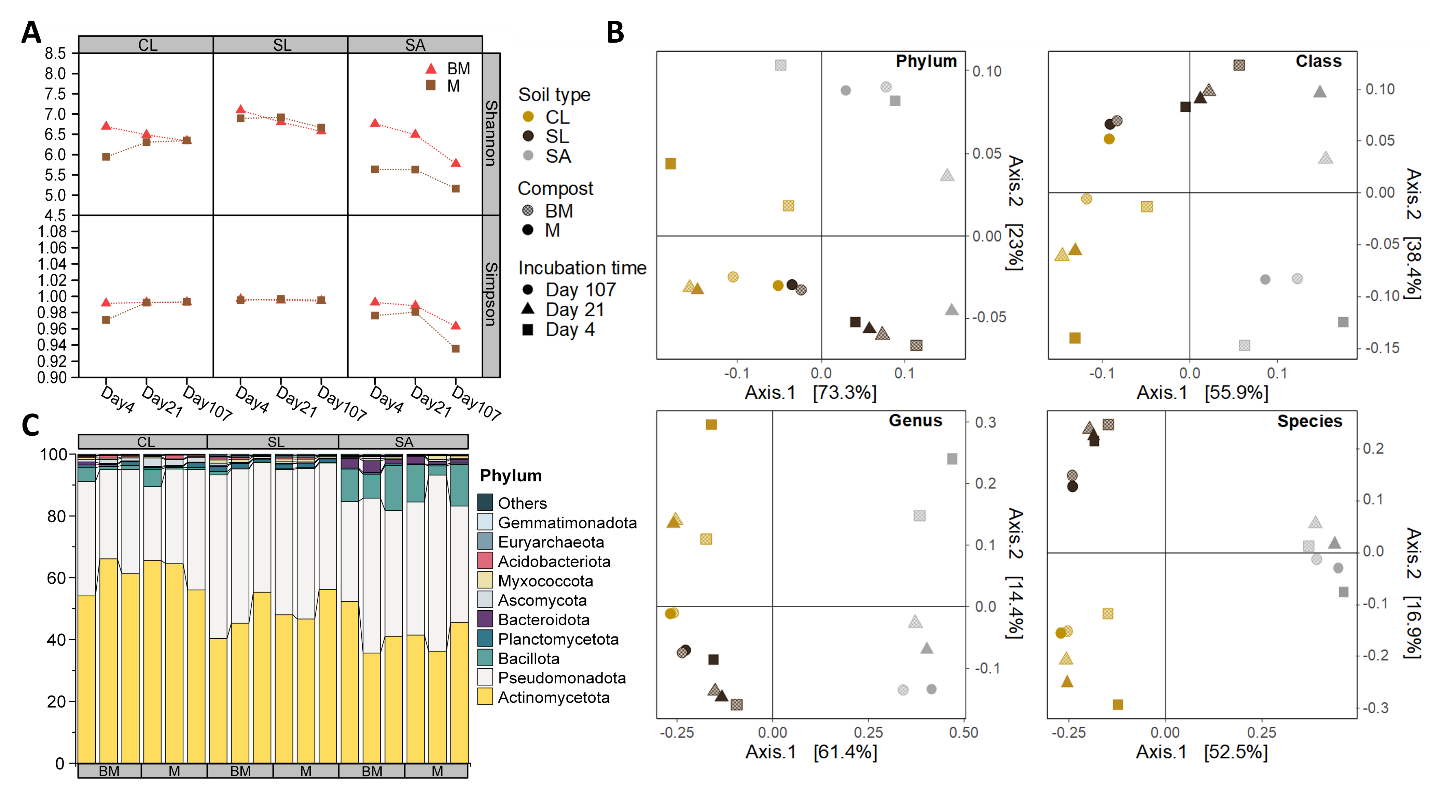


Fig. S2. Alpha diversity (**A**), beta diversity at different taxonomy levels (**B**), and phylum-level community composition (**C**) of microbial communities in clay loam (CL), silty loam (SL), and sand (SA) with the addition of BM (biochar-manure co-compost) and M (manure compost) at three time points (day 4, day 21, and day 107) during the 107 days’ incubation.


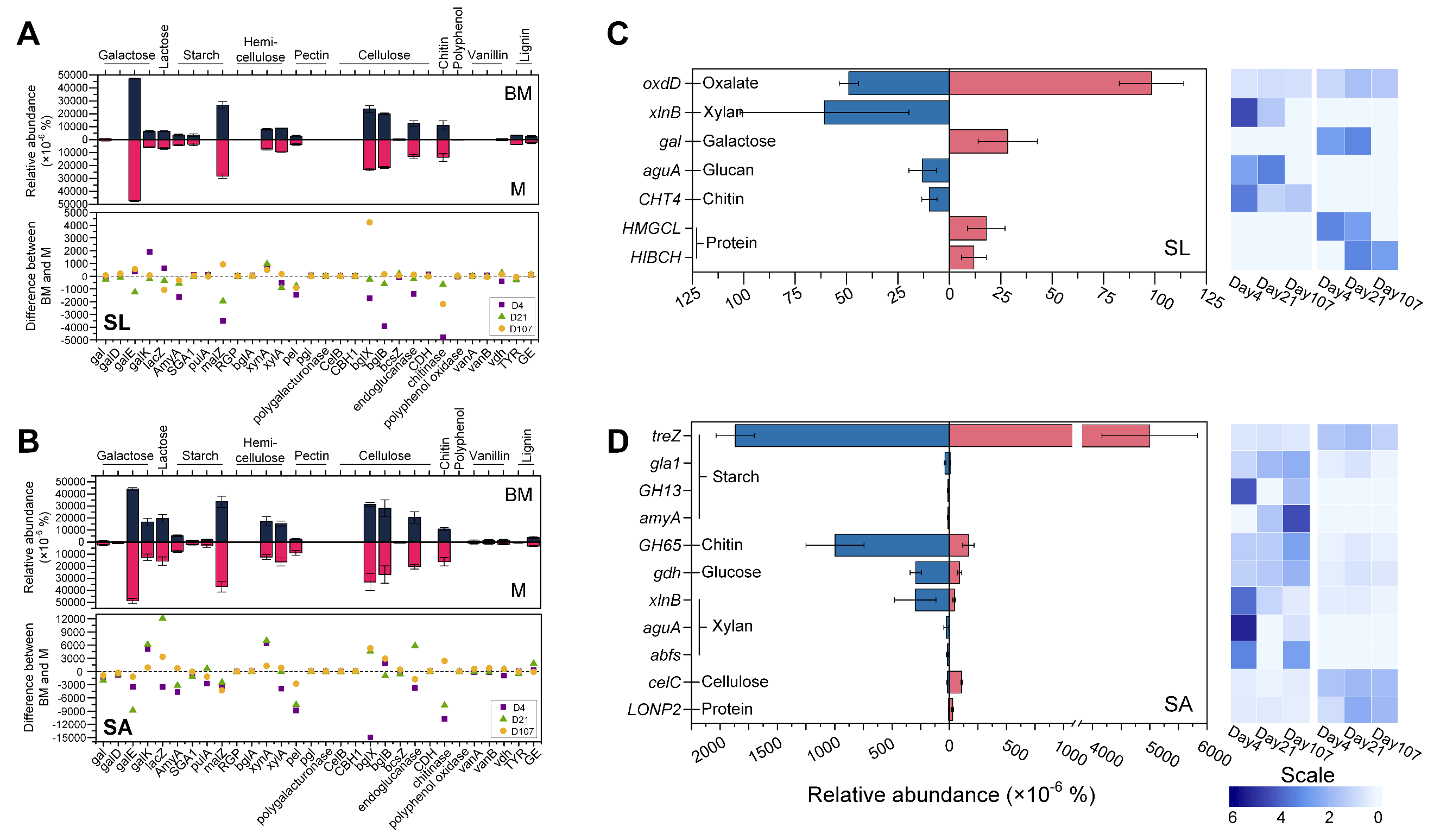


Fig. S3. Relative abundances of carbon-degradation genes in silty loam (SL), and sand (SA) based on KEGG orthology database (**A**, **B**) and eggnog ontology database (**C**, **D**), respectively, following the addition of BM (biochar-manure co-compost) and M (manure compost). The bar plots compare the difference between BM and M. Error bars are standard errors for n = 3. The scatter plots in (**A**, **B**) compare the difference between BM and M among three time points (day 4, day 21, and day 107) during the incubation period. The heatmaps in (**C**, **D**) show the relative abundance at three time points (day 4, day 21, and day 107) during 107 days’ incubation. Scale represents the normalized relative abundance by dividing the relative abundance by the average value across three time points.


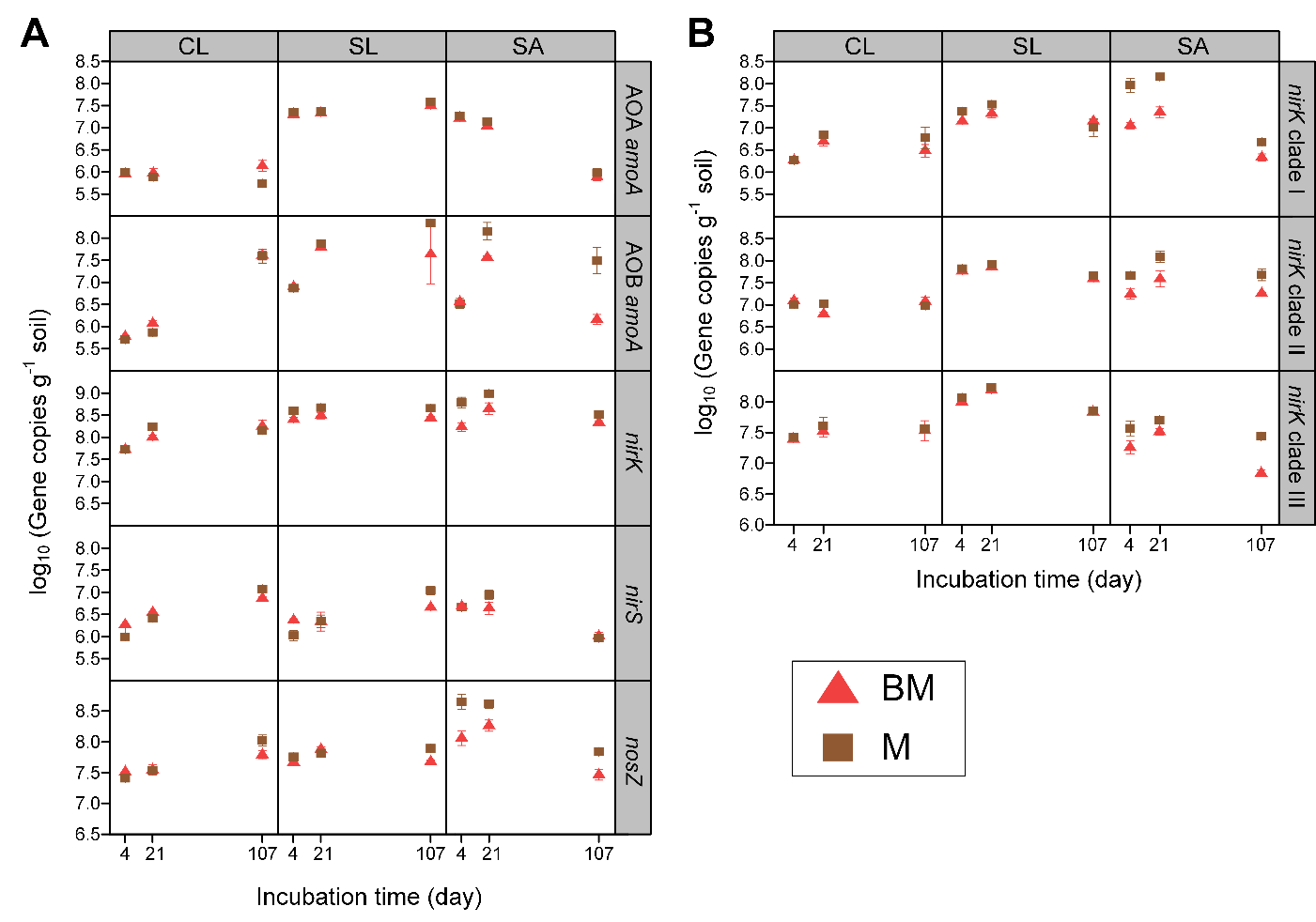


Fig. S4. qPCR-based absolute abundances of archaeal and bacterial *amoA*, prokaryotic *nirK*, *nirS*, and *nosZ* targeted by conventional primer sets (**A**) and different clades of prokaryotic *nirK* targeted by novel primer sets (**B**) at three time points (day 4, day 21, and day 107) during 107 days’ incubation following the addition of BM and M composts in clay loam (CL), silty loam (SL), and sand (SA). BM, biochar-manure co-compost; M, manure compost. Error bars are standard errors for n = 3.
